# Influence of the height of the mandibular ridge on the masticatory function during the functional adaptation with new complete dentures

**DOI:** 10.1590/1678-7757-2020-0092

**Published:** 2020-10-19

**Authors:** Vivian Barnabé POLICASTRO, Ana Flávia Balestrero CASSIANO, Marcela Dantas Dias da SILVA, Hamile Emanuella do Carmo VIOTTO, Andressa Rosa Perin LEITE, Danny Omar Mendoza MARIN, Ana Carolina PERO

**Affiliations:** 1 Universidade Estadual Paulista Faculdade de Odontologia de Araraquara Departamento de Materiais Odontológicos e Prótese Araraquara SP Brasil Universidade Estadual Paulista (UNESP), Faculdade de Odontologia de Araraquara, Departamento de Materiais Odontológicos e Prótese, Araraquara, SP, Brasil.; 2 Universidade Federal de Santa Catarina Faculdade de Odontologia Departamento de Odontologia Restauradora Florianópolis SC Brasil Universidade Federal de Santa Catarina, Faculdade de Odontologia, Departamento de Odontologia Restauradora, Florianópolis, SC, Brasil.

**Keywords:** Complete denture, Mastication, Bite force, Patient satisfaction

## Abstract

The period of functional adaptation to a new conventional complete denture embraces many transitory issues, and this period is directly related to the rehabilitation success.

**Objective:**

This clinical trial evaluated the influence of the height of mandibular ridge on the masticatory function of complete denture (CD) wearers during the adaptation period.

**Methodology:**

A total of 28 individuals wearing new CDs (NR, n=14, normal mandibular ridges, 64±12.5 years, 9 female; RR, n=14, resorbed mandibular ridges, 69±6.8 years, 9 female) were assessed at 24 hours, 30 days, three months and six months after the insertion of the CDs for masticatory performance (MP, sieves method), satisfaction with CDs (questionnaire) and maximum occlusal bite force (MOBF) (gnatodynamometer). The classification of the mandibular ridges followed the Kapur index. Data of MP and MOBF were analyzed by two-way ANOVA and satisfaction with CDs was analyzed by Generalized Estimating Equations (GEE), α=.05.

**Results:**

Participants with NR presented better masticatory performance (p=.000 - NR 30.25±9.93%, RR 12.41±7.17%), general satisfaction (p=.047), retention of mandibular denture (p=.001), chewing ability (p=.037), and comfort of wearing a mandibular denture (p=.000). Regardless of the mandibular ridge, MP (p=.000) was higher at three (21.26±12.07%) and six months (24.25±12.26%) in comparison to 24 hours (18.09±10.89%), the MOBF (p=.000) was higher at three months (78.50±6.49 N) compared to 24 hours (57.34±5.55 N) and 30 days (62.72±5.97 N), and the comfort of wearing a mandibular denture (p=.002) at three months (1.61 ± 0.07) was greater than 24 hours (1.29±0.10) and 30 days (1.36±10).

**Conclusions:**

The study suggests that the participants with NR have higher MP and satisfaction with their CD, regardless of the follow-up period after the insertion of the new CD. After subjects received the CD, a period of 3 months was necessary for achieving better achievement MOBF, MP, and self-perceived comfort with the mandibular denture, regardless of the height of the mandibular ridge.

## Introduction

Edentulism has been frequently observed worldwide for centuries.^1^ The rehabilitation with conventional complete denture (CD) is the first option for edentulous individuals because it is economically more viable than implant-supported dentures, thus a significant option for rehabilitation.^2,3^ Some studies have shown that treatment with conventional CD provides a high level of satisfaction among their users (65-90%),^3^ providing these patients with improved masticatory and phonetic functions, increased bite force, and quality of life.^4^

The period of functional adaptation begins after the patients receive the new conventional CDs. In this period, many problems, although transitory, may occur and they directly influence the rehabilitation success.^5^ Mandibular dentures are usually the greatest cause of discomfort and low satisfaction among denture wearers due to their poor retention and stability, hampering one’s adaptation.^6^ A hypothesis for this hampered adaptation is related to a lower mandibular area and the process of resorption of the alveolar ridge after extraction or tooth loss, which occurs with greater intensity in the mandible.^7^ Studies have already shown that advanced age and the use of drugs with xerostomic effect are factors that can influence the patients’ adaptation and consequently the number of returns after the insertion of these dentures.^8^ Thus, the post-insertion period of CDs should not be neglected by the professional, since it is directly related to the success or failure of treatment.

During functional adaptation with the new conventional CD, the masticatory performance (MP) is a relevant aspect, since it is an significant and deficient function for conventional CDs wearers. Several factors may influence the MP of conventional CDs wearers, such as age, sex, height and shape of the residual alveolar ridges, retention and stability of the complete denture, occlusal bite force, neuromuscular control, activity of mandibular muscles, salivary flow, occlusal scheme, posterior tooth forms, and previous experience with dentures.^9-11^ The masticatory performance of CD wearers is 10 to 20% in comparison to subjects with natural teeth.^12-15^

Another aspect of interest among conventional CDs wearers is the maximum occlusal bite force (MOBF) strength that these individuals can exert, which is related to their chewing ability.^16^ The MOBF is an objective evaluation of denture quality and performance, and it is closely related to masticatory function.^17^ A CD wearer can exert only 15% of the force of a subject with natural teeth.^11^ In the functional adaptation with new CDs, the MOBF increases progressively after the insertion of the new denture.^18^ The process of mandibular alveolar ridge resorption may directly influence the MOBF.^19^

Patients’ satisfaction with their new CD is extremely important to evaluate the treatment success, since the individual’s well-being indicates success in treatment.^20^ Studies have shown that different factors can influence patients’ satisfaction, such as the quality of the prosthesis, personality and psychological factors of the patients, salivary flow, age, sex, interaction between professional and patient, previous treatment experiences, cultural factors, and conditions of patients’ oral tissues, including the height of the mandibular ridge.^1,21,22^

The literature does not yet establish a relationship between the height of the mandibular ridge and physiological parameters and satisfaction of CDs wearers in the period after their installation. Only one study^23^ has presented, with subjective evaluation, that the degree of resorption of the mandibular alveolar ridge is a determining factor in the satisfaction of patients with new complete denture. However, the evaluation of objective variables was not carried out for this purpose, and other studies still seem to indicate a certain controversy about this problem.^24,25^

Thus, the aim of this study was to evaluate the influence of the height of the mandibular ridge in the MF, MOBF, and patient satisfaction at different stages after the insertion of a new set of conventional CDs. The primary outcome of this study is measurements of MP, six months after the insertion of the CD. Secondary outcomes include: MOBF and ratings of patient’s satisfaction during the 6-month follow-up period. The null hypothesis of this study was that the height of the mandibular ridges and the periods after the insertion of new conventional complete denture were not determinant factors on the MP, MOBF, and patient’s satisfaction.

## Methodology

This study is a longitudinal clinical trial with two-parallel groups and a follow-up period of six months. This study was approved by the Institutional Research Ethics Committee of Araraquara Dental School, Universidade Estadual Paulista, UNESP (CAAE Registry No. 55827216.8.0000.5416) and registered in the http://www.ensaiosclinicos.gov.br/ database with the identifier: RBR-98wb56.

Participants were recruited between June 2016 and December 2018 and the evaluations occurred in the six following months. Once participants provide six-month data, they were informed that they may contact the research team for unscheduled appointments if necessary.

### Participants

Potential participants were completely edentulous requiring replacement of the bimaxillary conventional complete denture who asked for treatment at the Department of Prosthodontics, School of Dentistry, Universidade Estadual Paulista (UNESP), Araraquara-SP, Brazil. The old dentures were evaluated by a prosthodontist, who attested for the replacement by new CDs, according to the following criteria: inadequate base extension or fit, incorrect maxillomandibular relationships, excessive wear of artificial teeth, cracks on denture base/teeth, and poor aesthetics.

The inclusion criteria for the study were: (a) patients capable of providing written informed consent, (b) good understanding of spoken Portuguese, (c) normal salivary flow (secretion of unstimulated saliva of 0.3-0.4 mL/min) ,^26^ (d) and complete denture wearers (at least one year). The exclusion criteria were: (a) participants who presented debilitating systemic conditions, pathological alterations of the alveolar ridges or disorders of the stomatognathic system, including signs/ symptoms of temporomandibular disorders.

Participants who fulfilled these criteria were assigned to the study groups (normal mandibular ridges or NR and resorbed mandibular ridges or RR) based on the classification described by the Kapur index^27^adapted by Gonçalves, et al.^16^(2014). According to the Kapur index,^27^ the mandibular ridges were classified considering their size and form; location of the inserted muscles and tissues and resilience of fibromucous. The size and form of the ridges were scored with a 4 point scale: 1 (“low or flat”), 2 (“V-shaped”), 3 (“shaped between U and V”), and 4 (“U-shaped”). The location of the inserted muscles and tissues were scored with a 3-point scale: 1 (high attachment), 2 (low attachment) and 3 (medium attachment). The resiliency of fibromucous of the ridge were scored with a 3 point scale: 1 (flabby), 2 (resilient) and 3 (firm). The mandibular ridges can scope a maximum score of 10 points. In this study, all participants were classified according to these scores and the individuals who presented scores equal to or higher than 7 were classified as having normal mandibular ridges (Group NR), whereas the resorbed mandibular ridges group presented scores lower than 7 (Group RR). The classification of the height of the mandibular ridges was performed by a single previously calibrated rater.

Individuals who fulfilled the characteristics of the inclusion and exclusion criteria were invited to participate in the study immediately after the insertion of the new conventional complete denture. A written consent form was obtained from the participants prior to the enrollment.

In this study, all dentures were fabricated according to the same clinical and laboratory procedures. The dentures were fabricated according to the conventional method described by Cunha, et al.^28^ (2013). A preliminary impression was obtained using stock trays and irreversible hydrocolloid (Jeltrate, Dentsply Industry and Commerce Ltd, Petrópolis, RJ, Brazil). Final impressions were made using acrylic resin trays and a polyether impression material (Impregum, 3M Espe, Sumaré, SP, Brazil). Maxillomandibular measures were recorded and the maxillar rim was transfered to the semi-adjustable articulator by a face-bow. The same dental technician fabricated all dentures using the same protocol. A heat-polymerized denture base-resin (Lucitone 550, Dentsply Industry and Commmerce Ltd, Petrópolis, RJ, Brasil) was used for the acrylization of the dentures according to the manufacturer’s instructions. All CD were fabricated with bilateral balanced occlusion (BBO) using 33-degree cusp angle acrylic resin teeth (Trubyte Biotone; Dentsply Industry and Commerce Ltd, Petrópolis, RJ, Brazil).

### Time points

The evaluations of MP, MOBF, and patient’s satisfaction with the complete denture were performed at different stages after the insertion of new conventional CDs: 24 hours (Evaluation 1),^7,29^ 30 days (Evaluation 2),^30,31^ three months (Evaluation 3) and six months after the denture insertion (Evaluation 4). Recall appointments were scheduled to adjust the CDs at anytime, if required, during the follow-up period. All the raters were blind for the alveolar ridge classification for the study variables.

### Masticatory performance (MP)

The MP was evaluated using the sieve method and some almonds were used as a natural test food.^27,32^ The participants were instructed to chew five almonds for 20 masticatory cycles. Chewing of the almonds was performed as usual, without swallowing any fragment, under the rater’s supervision. The comminuted particles were collected in a recipient, and the participant received 50 mL of water to rinse and remove the remaining particles which were also collected in the recipient. Then, the recipient was analyzed in a laboratory. The contents were poured into a sieve (sieve 1-7 cm, PLASTILLE, ref. 1188; dim.: 175 x 78 x 40 mm) adapted on a paper filter (no. 2 - Melitta Brazil’s Industry and Trade Ltd., Avaré, SP, Brazil) to separate the liquid from the chewed material, 500 ml of water were poured on the sieve to eliminate the saliva present in the almonds and to reduce particle clumping.

The crushed almonds were placed in an electric oven (Fanem Industry and Commerce Ltd, Guarulhos, SP, Brazil) at a controlled temperature of 130°C for 40 minutes, for dehydrating the material. The material was subjected to a 4-sieve system under constant vibration for 60 seconds in a gypsum vibrator. The sieves used (Granutest; Telastem Peneiras para Análise Ltda., Bom Retiro, São Paulo, SP, Brazil) were approved by the Brazilian Technical Standards Association (ABNT), and they had different hole sizes: 4.0 mm (ABNT 5), 2.8 mm (ABNT 7), 2.0 mm (ABNT 10), and 1.0 mm (ABNT 18). They were placed on top of each other, with the largest screen hole at the top and the smallest screen hole at the bottom, followed by a background collector to collect material that passed through the 4 sieves.

The total material (Pt) were weighed on a precision scale (Electric and Electronic Industry and Trade Gehaka Ltd., São Paulo, SP, Brazil), and this value was recorded in order to calculate the MP. The material retained in sieves 3 (hole sizes: 2.0 mm), 4 (hole sizes: 1.0 mm) and the background collector was weighed (P1). The masticatory performance was measured and expressed in percentage, according to the index proposed by Kapur and Soman^13^ (2006):


MP = P1 x 100/Pt

Where:

MP means masticatory performance (in percentage);

P1 represents the material weight sum in sieves 3, 4, and the background collector;

Pt represents the total material weight subjected to sieving.

### Maximum occlusal bite force (MOBF)

A digital gnatodynamometer IDDK model with a capacity of 100Kgf (Kratos Works Ltd, Cotia, São Paulo, SP, Brazil) was used to measure the MOBF. The instrument was positioned in the region of the first molars of the new conventional CDs. Participants sat upright in a dental chair in the orthostatic position, without head support and they were instructed to occlude the teeth by exerting their MOBF for 5 seconds, without pain or discomfort.^17,33,34^ The same procedure was repeated on the right and left sides with intervals of 30 second until three measurements were carried out in each side. The values obtained were recorded in Newton (N) and a mean was estimated for each experimental condition.

### Patient satisfaction

Participants answered a denture satisfaction questionnaire based on the criteria used by Celebic and Knezovic-Zlataric^35^ (2003) and described by Souza, et al.^36^ (2012). This questionnaire addresses questions related to general satisfaction, comfort of wearing maxillary and mandibular dentures, aesthetics, retention of maxillary and mandibular dentures, and the ability to speak and chew with the new conventional complete dentures. Possible answers for each question and the respective scores were: (A) unsatisfactory (‘0’); (B) regular (‘1’); (C) good (‘2’). Higher scores represent better satisfaction with the complete denture, and this result may vary from 0 to 16. The questionnaire was applied by a rater who had been blinded to the mandibular ridge classification.

### Sample size

The sample size was estimated based on previous pilot study, considering the minimum significant difference for MP of 17 and a standard deviation of 15.2 between the study groups (NR and RR). This estimation was carried out from a convenience sample of seven patients for both groups. Considering a power analysis of 80% and α=0.05, 14 individuals were necessary for each group.

### Statistical analysis

The assumptions of the method (normality, equal variances, and sphericity) were verified by the Shapiro-Wilk (p>0.05), Levene (p>0.05), and Mauchly’s (p>0.05) tests, respectively.

Data on MP (%) and MOBF (N) were analyzed by the two-way repeated-measures ANOVA (mixed design). The patient satisfaction with the new CD was analyzed by generalized estimating equations (GEE). This method of data analysis was considered as adequate for the measures collected within the different evaluated periods presented no homogeneity of variances or independence.^37,38^

These analyses considered two factors: the height of the mandibular ridges (NR or RR) and follow-up periods after the insertion of new conventional CDs (24 hours, 30 days, three months, and six months). Statistical analyses were performed using the PASW Statistics software, version 19 (SPSS Inc., Chicago, IL, USA) with a 5% significance level. The statistician was blind for the study groups.

## Results

### Participants

**Figure 1:**
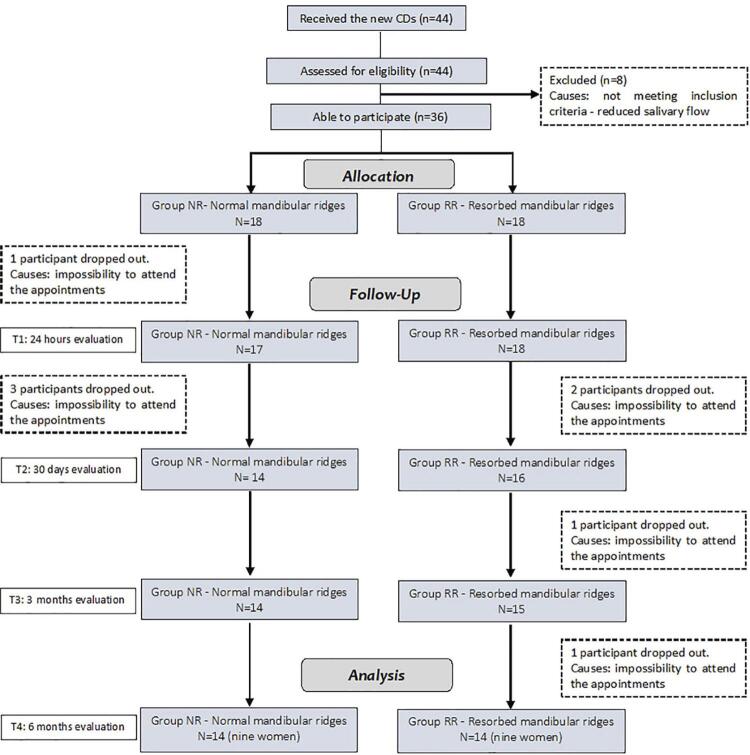
Flowchart of participants

Figure 1 shows a flowchart of the participants in the present clinical trial. Table 1 shows the sociodemographic characteristics of the participants for each group. In total, 44 edentulous patients (30 women and 14 men) were assessed for participation in the study and received a new set of complete dentures. A total of 36 individuals that fulfilled the inclusion and exclusion criteria were enrolled and assigned to the study groups: NR group (n=18) or the RR group (n=18). Twenty-eight participants completed the study.

**Table1:** Clinical characteristics of the participants for each group

Characteristic		Group NR (n=14)	Group RR (n=14)
		Mean (±SD*)	Mean (±SD*)
Age (years)		64±12.5 years	69±6.8 years
Gender (female/male)		09/05	09/05
Birthplace in Brazil	Southeast region	14	14
Lives with (Family/Alone)		10/04	11/03
	Illiterate	0	0
Instruction	Elementary school	5	9
	High school	9	5
	Married	9	7
Civil Status	Single	3	3
	Widower	1	2
	Divorced	1	2
	Retired	6	9
Profession	Unemployed	1	2
	Employee / Self Employed	7	3
Mean edentation time (years)		27±14.3 years	30±12.15 years
Mean time of wearing CD (years)		25±13.4 years	28±10.91 years
Score of Kapur index		7.57	4.57
Salivary flow (mL/min)		0.62±0.3	0.42±0.27

* SD: standard deviation

### Masticatory performance (MP)

The two-way ANOVA detected that the mandibular ridge height (*p*=0.000) and the follow-up periods were significant factors for the outcome variable (*p*=0.000). There was not significant effect of the interaction between mandibular ridge x follow-up periods (*p*=0.052).

**Figure 2:**
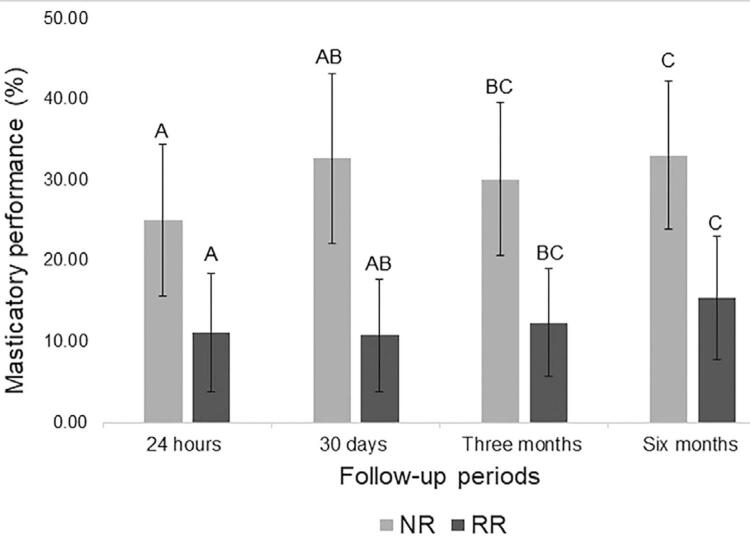
Multiple comparisons of masticatory performance (Bonferroni’s test, p<0.05) Different letters represent statistically significant differences between periods (p<0.05)

Participants with NR (30.25±9.93%) had better MP than RR (12.41±7.17%), regardless of the follow-up period. Moreover, Figure 2 shows that, regardless of the mandibular ridge height, a significant improvement of MP (p=0.000) was observed at three months (21.26±12.07%) and six months (24.25±12.26%), in comparison to the 24 hours after the insertion of the complete dentures (18.09±10.89%).

### Patient satisfaction

Table 2 shows the results for the satisfaction questionnaire with the new conventional complete dentures. This table presents mean scores and standard deviations for each question (Q1 to Q8), according to the group and follow-up period, and the p values for the factors “ridge,” “period,” and the interaction “ridge x period.” It was observed that the “mandibular ridge” factor was significant for questions about the general satisfaction (p=0.047), retention of the mandibular CD (p=0.001), chewing ability (p=0.037) and the comfort of wearing a mandibular denture (p=0.000). Regardless of the period, participants with NR significantly improved these aspects compared to RR. For the question related to the comfort with the mandibular denture (Q8), the factor “period” was also significant (p=0.002). It means that participants with NR had more comfort with their mandibular dentures compared to RR, and a three-month period was necessary for this achievement, regardless of the height of the mandibular ridge. The height of the mandibular ridges and follow up periods were not significant for denture aesthetics, ability to speak and comfort of wearing a maxillary denture.

**Table2:** Mean scores (±standard deviation) of the patient’s satisfaction questionnaire

Satisfaction questionnaire	Normal mandibular ridges				Resorbed mandibular ridges				GEEs, p-valor		
	24 hours	30 days	3 months	6 months	24 hours	30 days	3 months	6 months	Ridge	Periods	Interaction
Q1 (general satisfaction)	2 (2-2)	1.93 (1.79-2.06)	1.93 (1.79-2.06)	1.86 (1.59-2.13)	1.86 (1.59-2.13)	1.79 (1.57-2)	1.71 (1.48-1.95)	1.5 (1.17-1.83)	0.047*	0.189	0.565
Q2 (retention of maxillary denture)	1.79 (1.57-2)	2 (2-2)	2 (2-2)	2 (2-2)	1.93 (1.79-2.06)	1.64 (1.32-1.96)	1.93 (1.79-2.06)	1.71 (1.41-2.02)	0.108	0.028	0.015*
Q3 (retention of mandibular denture)	1.93 (1.79-2.06)	1.71 (1.48-1.95)	1.86 (1.67-2.04)	1.79 (1.57-2)	1.43 (1.1-1.75)	1.21 (0.81-1.62)	1.14 (0.75-1.53)	1.07 (0.65-1.49)	0.001*	0.057	0.680
Q4 (aesthetics)	1.93 (1.79-2.06)	2 (2-2)	1.93 (1.79-2.06)	1.93 (1.79-2.06)	2 (2-2)	1.93 (1.79-2.06)	2 (2-2)	1.86 (1.67-2.04)	1000	0.333	0.333
Q5 (ability to speak)	1.93 (1.79-2.06)	1.86 (1.67-2.04)	2 (2-2)	2 (2-2)	1.64 (1.32-1.96)	1.71 (1.41-2.02)	1.71 (1.41-2.02)	1.57 (1.19-1.95)	0.059	0.233	0.123
Q6 (chewing ability)	1.36 (1.11-1.61)	1.57 (1.25-1.90)	1.64 (1.32-1.96)	1.64 (1.32-1.96)	1 (0.66-1.34)	1.14 (0.81-1.48)	1.07 (0.76-1.38)	1.36 (0.93-1.78)	0.037*	0.140	0.444
Q7 (comfort of maxillary denture)	1.86 (1.67-2.04)	2 (2-2)	2 (2-2)	2 (2-2)	1.86 (1.59-2.13)	1.79 (1.57- 2)	1.93 (1.79- 2.06)	1.79 (1.49- 2.08)	0.108	0.269	0.105
Q8 (comfort of mandibular denture)	1.64 (1.39-1.89)	1.50 (1.24-1.76)	1.86 (1.67-2.04)	1.86 (1.67-2.04)	0.93 (0.62-1.24)	1.21 (0.92-1.51)	1.36 (1.11-1.61)	1.29 (0.92- 1.65)	0.000*	0.002*	0.281

*significant difference between groups (p<0.05). Minimum possible value: 0; maximum possible value: 2

**Figure 3:**
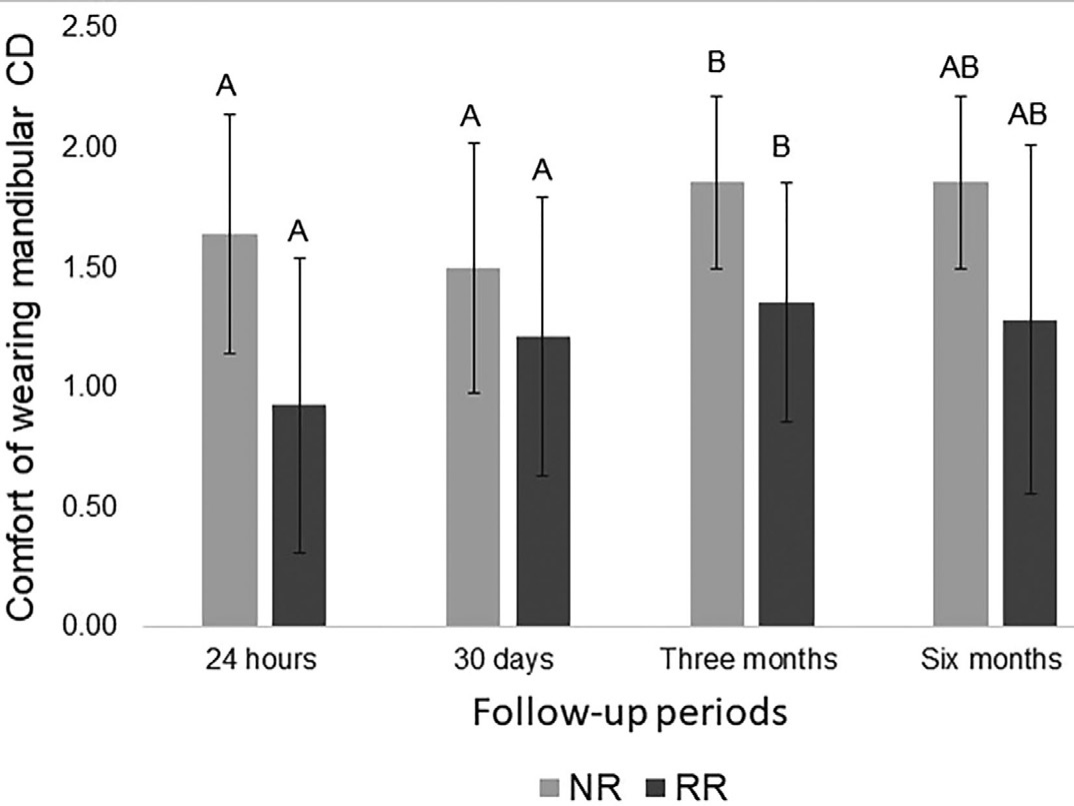
Multiple comparison of means (±standard deviation) on comfort of wearing a mandibular complete denture (Bonferroni’s test, p<0.05) Different letters represent statistically significant differences between periods (p<0.05).

Figure 3 shows a significant improvement of the comfort of wearing a mandibular CD (p=0.002), irrespective of the height of the mandibular ridge, at three months (µ=1.61±0.079) in comparison to the 24 hours (µ=1.29±0.10) and 30 days after the denture insertion (µ=1.36±0.1).

Moreover, there was a significant effect of the interaction between the mandibular ridge x follow-up period factors for the retention of the maxillary CD (p=0.015). Table 3 shows that at 30-day follow-up period, participants with NR (2.00±0.00) had a higher satisfaction with the retention of the maxillary CD than participants with RR (1.64±0.16).

**Table 3:** Multiple comparison of means (±standard deviation) for the maxillary complete denture retention (Bonferroni’s test, p<0.05)

	24 hours	30 days	3 months	6 months
Normal mandibular ridges	1.79±0.11^Aa^	2.00±0.00^Aa^	2.00±0.00^Aa^	2.00±0.00^Aa^
Resorbed mandibular ridges	1.93±0.07^Aa^	1.64±0.16^Ab^	1.93±0.07^Aa^	1.71±0.16^Aa^

Different capital letters in line indicate statistically signiﬁcant differences; similar lowercase letters in columns indicate similarity.

### Maximum occlusal bite force (MOBF)

The two-way ANOVA detected that the follow-up periods were significant for the outcome variable (*p*=0.000). The resorption height of the mandibular ridge (*p*=0.082) and the interaction mandibular ridge x follow-up periods (*p*=0.566) was not significant.

**Figure 4:**
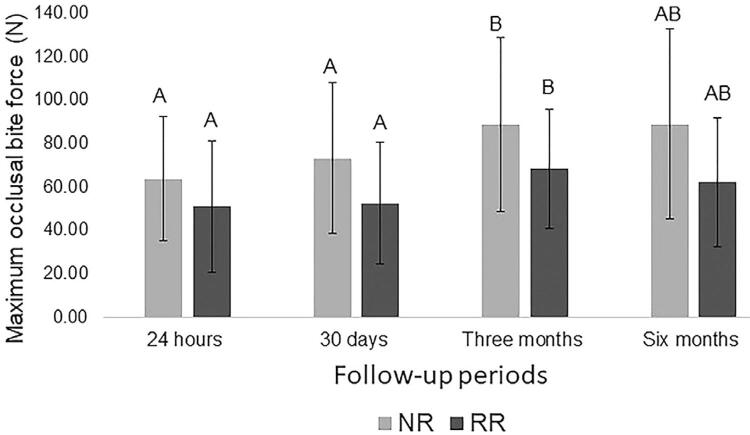
Multiple comparison of maximum occlusal bite force means (Bonferroni’s test, p<0.05) Different letters represent statistically significant differences between periods (p<0.05).

Figure 4 shows a significant improvement of MOBF (*p*=0.000). The MOBF was higher at three months (78.50±٦.٤٩N) in comparison to the 24 hours (57.34±5.55N) and 30 days after the denture insertion (62.72±5.97 N), regardless of the mandibular ridge height.

## Discussion

The null hypothesis of this study was rejected since the height of the mandibular ridges and the follow-up periods after the new dentures insertion significantly influenced the outcomes of the patient with their dentures. We tested the hypothesis that the height of the mandibular ridges would influence the adaptation period with new CDs. Thus, we evaluated MP, MOBF, and patients’ satisfaction with their dentures.

Objective masticatory function (MP) is measured by determining the individual’s capacity to grind a test food after a fixed number of chewing cycles.^12,32,39^ It was observed that participants with NR presented better MP compared to participants with RR. Gonçalves, et al.^16^ (2014) carried out a study to observe the influence of the height of the mandibular ridge on mandibular movements during chewing of CD wearers and they observed significant changes in patients with resorbed mandibular ridges, indicating impairment in chewing function of these individuals. Pero, et al.^34^ (2019) evaluated the MP of CD wearers with different occlusal schemes and concluded that, regardless of the occlusal scheme, participants with NR presented better masticatory performance compared to participants with RR. Furthermore, our results of MP are related to the satisfaction of these participants with RN who stated better retention and comfort with the mandibular dentures and better self-perception of chewing. Thus, our results reinforce the premise that retention and comfort are crucial factors for better masticatory performance, regardless of the follow-up period.

This study demonstrated that the height of the mandibular ridge influenced the self-perception of general satisfaction, the retention of the mandibular complete denture, the chewing ability and the comfort of wearing a mandibular denture as better for participants with normal mandibular ridges, regardless of the follow-up period. The height of the mandibular ridge is strongly related to the stability and retention problems of the mandibular complete denture,^21-23^ causing discomfort during the function. Moreover, the mucosa overlying the resorbed ridges is usually more susceptible to ulcerations, and thus, the mastication process and the comfort of wearing a mandibular denture may be painful and limited for complete dentures wearers with resorbed denture-bearing areas.^40,41^

Our results are in agreement with Yamaga, et al.^22^ (2013). These authors found that patients with NR were more satisfied with the conventional mandibular CD in comparison to the RR ones. Fenlon and Sherriff^23^(2008) observed that patients with RR mostly complained about the stability and retention of their mandibular CDs. Comfort, stability, and retention of the CD are determining factors for the chewing ability.^42^ In our study, the results of the satisfaction questionnaire showed that the participants with RR had poor self-perceived capacity to chew and discomfort with their mandibular CDs.

The results of our study demonstrated that participants at the three months follow-up had better self-perception about their comfort of wearing a mandibular denture than participants at 24 hours and 30 days, regardless of the height of the mandibular ridge. Regis, et al.^43^ (2013) concluded that six months were necessary for patients be satisfied with their new conventional CDs and to relieve any initial complaints, however, the participants did not receive returns for CD adjustments during the three-month and six-month evaluations, which may justify the results contrasting with our study. In our study, after the insertion of the new CDs, adjustments recalls were scheduled if asked by the participants, which may have resulted in a shorter time to feel comfortable with their complete dentures and a faster functional adaptation process.

In this study we also observed that, within 30 days after the insertion of conventional complete dentures, patients with NR had better satisfaction with the retention of the maxillary denture than patients with RR. A hypothesis for this finding is that, at 30 days, patients with RR did not have coordinated mandibular movements, resulting in diffused occlusal forces, possibly altering the retention of the maxillary CD. Previous studies support this hypothesis,^44^ in which the authors state that when a CD is subjected to occlusal forces, the supporting tissues may deform and, consequently, the complete denture displaces, which may affect patients’ self-perception regarding the retention of the CD. In the following periods of this study, there was no difference regarding the retention of the maxillary CD, indicating that patients could possibly be more coordinated, distributing the occlusal forces better, resulting in better self-perception due to the retention of their maxillary CDs.

The results of this study showed that participants at three and six months after the insertion of the new CDs had better MP in comparison to the 24 hours-period, regardless of the height of the mandibular ridge. These results agree with Eberhard, et al.^45^ (2018) who observed that after the insertion of new conventional complete dentures, patients’ Masticatory performance gradually improved until the follow-up period of three months. It has been stated that the stomatognathic system gradually adapts to new CDs, resulting in better MP due to the patient’s adaptation to occlusion, stability, retention, and comfort with their dentures.

The follow-up period also had an influence on the MOBF in this study. Three months after the insertion of new CDs, participants presented higher values of bite force in comparison with the 24 hour and 30 day-periods, regardless of the height of the mandibular ridge. In the study of Shala, et al.^46^ (2018), it was observed that the MOBF was reached within 15 weeks after the insertion of new CDs, remaining stationary after this period. The period to reach a steady state varies greatly, ranging between 12 and 24 months.^47^ Moreover, it is noticeable that, in this study, participants at three months felt more comfortable with the mandibular denture, which could explain the higher MOBF over the same period.

The limitations of this longitudinal clinical trial include the sample characteristics. The results should not be extrapolated to patients with other conditions (for example; low salivary rate or patients who had never used a complete denture). It could be hypothesized that patients with low salivary rate would present poorest MP, since saliva plays a significant role during the motor activities like chewing and swallowing.^39,48^ Furthermore, different results might be addressed if participants had been previously experienced complete dentures.^1,21,22^

Few participants were lost during the follow-up, and the groups were equal in number of male and female individuals. Moreover, the groups were homogeneous regarding age and only patients with normal salivary flow were included to avoid bias.

Further studies are necessary to clinically evaluate other aspects involving the adaptation period with the new conventional CD, and a more long-term follow-up study.

## Conclusion

Therefore, this longitudinal clinical trial demonstrated that, regardless of mandibular ridge height, a three-month period after the insertion of the new dentures is necessary to improve MP, MOBF, and the comfort of wearing mandibular CD. It was also demonstrated that individuals with normal mandibular ridges have better masticatory performance and they are more satisfied during the adaptation period with new conventional complete dentures.

In summary, this study could be helpful to the clinical professionals, which should be careful with the adjustments, maintenance and instructions up to three months after the insertion of new conventional complete dentures, regardless of the height of the mandibular ridge.
